# Cost of Fetal Alcohol Spectrum Disorder Diagnosis in Canada

**DOI:** 10.1371/journal.pone.0060434

**Published:** 2013-04-04

**Authors:** Svetlana Popova, Shannon Lange, Larry Burd, Albert E. Chudley, Sterling K. Clarren, Jürgen Rehm

**Affiliations:** 1 Social and Epidemiological Research Department, Centre for Addiction and Mental Health, Toronto, ON, Canada; 2 Dalla Lana School of Public Health, University of Toronto, Toronto, ON, Canada; 3 Factor-Inwentash Faculty of Social Work, University of Toronto, Toronto, ON, Canada; 4 Institute of Medical Sciences, University of Toronto, Toronto, ON, Canada; 5 Department of Pediatrics, University of North Dakota School of Medicine and Health Sciences, Grand Forks, North Dakota, United States of America; 6 Children’s Hospital, Health Sciences Centre, Departments of Pediatrics and Child Health and Biochemistry and Medical Genetics, University of Manitoba, Winnipeg, MB, Canada; 7 The Canada Fetal Alcohol Spectrum Disorder Research Network, Centre for Community Child Health Research, The Child and Family Research Institute of British Columbia, Vancouver, BC, Canada; 8 Department of Paediatrics, University of British Columbia Faculty of Medicine, BC Children’s Hospital, Vancouver, BC, Canada; 9 School of Medicine, University of Washington, Seattle, Washington, United States of America; 10 Epidemiological Research Unit, Klinische Psychologie and Psychotherapie, Technische Universität Dresden, Dresden, Germany; Groningen Research Institute of Pharmacy, United States of America

## Abstract

**Background:**

Fetal Alcohol Spectrum Disorder (FASD) is underdiagnosed in Canada. The diagnosis of FASD is not simple and currently, the recommendation is that a comprehensive, multidisciplinary assessment of the individual be done. The purpose of this study was to estimate the annual cost of FASD diagnosis on Canadian society.

**Methods:**

The diagnostic process breakdown was based on recommendations from the Fetal Alcohol Spectrum Disorder *Canadian Guidelines for Diagnosis*. The per person cost of diagnosis was calculated based on the number of hours (estimated based on expert opinion) required by each specialist involved in the diagnostic process. The average rate per hour for each respective specialist was estimated based on hourly costs across Canada. Based on the existing clinical capacity of all FASD multidisciplinary clinics in Canada, obtained from the 2005 and 2011 surveys conducted by the Canada Northwest FASD Research Network, the number of FASD cases diagnosed per year in Canada was estimated. The per person cost of FASD diagnosis was then applied to the number of cases diagnosed per year in Canada in order to calculated the overall annual cost.

**Results:**

Using the most conservative approach, it was estimated that an FASD evaluation requires 32 to 47 hours for one individual to be screened, referred, admitted, and diagnosed with an FASD diagnosis, which results in a total cost of $3,110 to $4,570 per person. The total cost of FASD diagnostic services in Canada ranges from $3.6 to $5.2 million (lower estimate), up to $5.0 to $7.3 million (upper estimate) per year.

**Discussion:**

As a result of using the most conservative approach, the cost of FASD diagnostic services presented in the current study is most likely underestimated. The reasons for this likelihood and the limitations of the study are discussed.

## Introduction

Prenatal alcohol exposure (PAE) can lead to a myriad of adverse developmental outcomes, and is recognized to be the most common preventable cause of mental deficiency in the western world. Despite attempts to increase public awareness of the risks associated with drinking during pregnancy, a significant proportion of pregnancies among the general population (about 14%) [1] and among aboriginal populations (about 50% in Alberta in an isolated Northern community [2] and more than 60% among Intuits from Arctic Quebec [3]) in Canada are alcohol-exposed.

Fetal Alcohol Spectrum Disorder (FASD) is a non-diagnostic term that encompasses four alcohol-related clinical diagnoses, including: Fetal Alcohol Syndrome (FAS), Partial Fetal Alcohol Syndrome (pFAS), Alcohol-Related Neurodevelopmental Disorder (ARND), and Alcohol-Related Birth Defects (ARBD). The effects of PAE may include growth impairments (height and weight), birth defects, neuropsychiatric disorders, and mental disorders (neurobehavioural and/or learning disabilities), which are likely to have lifelong implications.

Given the wide range of deficits and disabilities associated with FASD, it is absolutely necessary to screen for individuals who may be affected by PAE. This is especially important for ARND since these disorders are not apparent from physical features [4]. The recognition of FASD is important for an individual at any age. Early screening may lead to early diagnosis, which can lead to early participation in developmental interventions, which can in turn, improve the quality of life for people with an FASD. Early intervention may also increase the potential for the prevention of secondary disabilities including school failure and drop-out, addictions, mental health problems, sexually deviant behaviour, dependent living, involvement with the law, and incarceration [5], [6]. Having an official diagnosis of FAS appears to have long-term benefits for people with an FASD. Early diagnosis and providing an appropriate environment improves outcomes and decreases the risk for additional impairments by up to four fold (e.g., Paintner et al. [7]). Early diagnosis is also important for parents/caregivers. It provides them with an explanation for the behavioural problems often exhibited by children with FASD and can improve parenting by increasing their understanding of the individual disabilities and impairments.

Importantly, screening of children for PAE and early diagnosis can also facilitate the prevention of subsequent FASD-affected births by providing appropriate interventions, treatment, counseling, and support for birth mothers with unrecognized alcohol dependence and mental health problems [8]. Appropriate screening strategies may also facilitate early recognition and intervention for other affected siblings [9].

In 2005, the Public Health Agency of Canada (PHAC) endorsed the Canadian Guidelines for the diagnosis of FASD [10]. According to these guidelines, the full diagnostic process involves screening and referral, the physical examination (including the dysmorphology assessment), the neurobehavioural assessment, differential diagnosis, and confirmation of PAE (with exception of an FAS diagnosis, which can be diagnosed without confirmation of PAE).

### Screening and Referral for FASD

Screening is a process by which members of a defined population are asked a question or offered a test to determine which of those individuals are more likely to be helped by further tests/assessments. In the case of FASD, ideally, a positively screened individual will undergo a diagnostic evaluation for FASD. It is important to understand that not all positively screened individuals will receive an FASD diagnosis. The purpose of FASD screening is to facilitate referral to a diagnostic clinic, and to call attention to the need for referral and support for the birth mother [10].

Despite having the most commonly cited rough prevalence estimates of 1 per 1,000 for FAS [11] and 9 per 1,000 for FASD [12], there is currently no widely accepted standardized FASD screening test in Canada [13]. One of the reasons for this is that the validity and reliability of screening tools have not yet been verified. However, it should be recognized that great strides have been made in regard to screening tool research and development in Canada. In a review and evaluation of published and practiced methods of screening, Goh et al. [13] selected five tools to be included in an FASD screening toolkit: screening fatty acid ethyl esters (FAEE) in neonatal meconium, the modified Child Behaviour Checklist, Medicine Wheel tool, Asante Centre Probation Officer Tool [14], and maternal history of drinking and drug use. Tool selection was based on the tools’ feasibility and test characteristics (sensitivity, specificity, positive and negative predictive values). Further, the Canadian Association of Paediatric Health Centres (CAPHC) facilitated the development of a national screening toolkit for children and youth identified and potentially affected by FASD [15]. This toolkit (containing many of the tools selected by Goh et al. [13]) was officially launched in October 2010, and includes the Neurobehavioural Screening Tool, Meconium FAEE Testing, Maternal Drinking Guide, Medicine Wheel tool, and the Asante Centre Probation Officer Tool [14].

It is also important to understand that the screening tools utilized in a population of children and youth may not be applicable to a population of adults and thus, tools should be age appropriate. Some tools have been recommended specifically for use in adults [16].

Screening is only effective if the right individuals are being screened. In regard to FASD, there are populations of special concern that should receive special attention in regard to the administration of screening for FASD, including (in no particular order):

- Women utilizing maternal substance abuse treatment programs
- Children seen in neurodevelopmental and/or birth defect clinics
- Children in special education programs
- Children in childcare systems (e.g., orphanages, foster care, child welfare/social services, etc.)
- Prison populations
- Youths in juvenile justice programs
- Clients of the mental health care system

The role of the family physician or primary care health provider in screening for FASD is to recognize patients with dysmorphic features and/or developmental/learning and behavioural problems that may be due to PAE, confirm the alcohol history, and then refer the patient to a multidisciplinary diagnostic team with expertise in FASD diagnosis [17]. However, similar screening could, and should, also be conducted through the educational system, the mental health system, the judicial system, and social services. It is important to point out that screening does not equate to and should not be used in lieu of a full diagnostic assessment [10].

### FASD Diagnosis

The diagnosis of FASD is not simple owing to the fact that determining if a patient has diffuse brain damage as an explanation for behavioural problems and that this damage is more likely due to PAE and not to other causes requires a comprehensive, multidisciplinary assessment involving physicians (generally developmental pediatricians, dysmorphologists and/or clinical geneticists, psychiatrists or neurologists), psychologists or neuropsychologists, joined by other developmental specialists (e.g., speech or language pathologists, occupational therapists and physiotherapists), as well as a coordinator for case management [10], [18], [19].

The criteria for FASD diagnoses have been described thoroughly in the current literature [10], [19]–[21].

FASD diagnosis involves the following components: the physical examination, the dysmorphology assessment, the neurobehavioural assessment, and PAE confirmation. The general physical examination involves appropriate measurements of growth (height, weight, and head circumference), assessment of characteristic findings and documentation of anomalies (for example, congenital heart defects, abnormal palmar creases, high arched palate). While, the dysmorphology assessment is intended to identify specific facial features related to PAE (e.g., length of palpebral fissure, philtrum smoothness, and upper lip [vermilion border] thinness). The neurobehavioural assessment involves an evaluation of multiple domains including: hard and soft neurological signs, brain structure, cognition (IQ), communication (receptive and expressive), academic achievement, memory, executive functioning and abstract reasoning, and attention deficit/hyperactivity. The specific diagnostic tests/evaluations will differ depending on the age of the individual (e.g., children versus adults) [16].

Confirmation of PAE (i.e., confirmation of alcohol consumption by the mother during the pregnancy in question) is also required for FASD diagnosis (with the exception of an FAS diagnosis, as stated above) and can be obtained through a direct interview with the mother (i.e., self-reports), or other sources such as reliable clinical observation, reports by a reliable source, or medical records [10], [20], [21].

Based on the information presented above, FASD diagnosis can easily be seen as a lengthy process that requires a number of trained specialists. Therefore, it is understandable that FASD diagnosis, like other complex brain based developmental disorders, is likely a very costly practice.

The purpose of this study was to estimate the per person cost of FASD diagnosis, as well as the annual cost of FASD diagnostic services in Canada.

## Methods

### Ethics Statement

Not applicable.

### Cost of FASD Diagnosis per Person

The diagnostic process breakdown was based on the Fetal Alcohol Spectrum Disorder: Canadian Guidelines for Diagnosis [10].

The number of hours required by each specialist involved in the diagnostic process was estimated, based on experts’ opinion (Larry Burd, Albert Chudley, Mary Cox-Millar, Gideon Koren, Sally Longstaffe, Kelly Nash, and Shelley Proven). The average rate per hour for each respective specialist was estimated based on hourly costs across Canada.

### Cost of All FASD Cases Diagnosed per Year

In order to estimate the annual cost of all FASD cases diagnosed for Canada as a whole, it is necessary to know how many cases are referred, evaluated and diagnosed per year in Canada. Unfortunately, these figures are not available. Therefore, the number of FASD cases diagnosed per year in Canada was estimated based on the data obtained from the 2011 survey conducted by the Canada Northwest FASD Research Network to determine the existing clinical capacity (the maximum number of assessment slots available for diagnostic purposes per year) of all FASD multidisciplinary clinics in Canada in 2011 [22]. The assumption that at most, about 70% (used as the upper boundary) of all individuals referred and evaluated are actually diagnosed with FASD in Canada was based on the data obtained from the 2005 survey, conducted by the Canada Northwest FASD Research Network among 27 FASD multidisciplinary clinics in Northwest Canada (response rate 56%). The survey revealed that among 816 evaluations in 2005, about 23% of those assessed were found to have FAS or pFAS and another 44% had other forms of FASD [18].

Fifty percent was then used as the lower boundary, in order to calculate the minimum number of people diagnosed with FASD in Canada per year (also based on the clinical capacity of Canada).

The cost of FASD diagnosis per person was applied to the number of cases diagnosed per year in Canada. All cost figures are presented in Canadian dollars.

## Results

### Cost of FASD Diagnosis per Person

The estimated number of hours, average cost per hour, and total cost for each component of the multidisciplinary diagnostic process are presented in Table 1. *Screening and Referral* was estimated to cost from $150 to $300 per person screened (1–2 hours per person), *Intake* into the diagnostic clinic, the point at which information is gathered, was estimated to cost from $160 to $320 (2–4 hours per person), *Diagnosis* was estimated to cost between $2,650 to $3,750 per person (23 to 33 hours per person), and *General Support* was estimated to cost between $150 to $200 per individual (6 to 8 hours per person). Thus, the total cost for one individual to be screened, referred, admitted, and diagnosed ranges from $3,110 to $4,570 (32 to 47 hours per person, in total).

**Table 1:** The per person cost estimate for each service involved in the multidisciplinary FASD diagnostic process.

Services	Involved specialists	Average number of hours	Average cost per hour	Average cost for total number of hours
**Screening and referral**	Physician/pediatrician/family doctor/social worker/probation officer*	1–2	$150	$150–$300
**Intake**	Coordinator/social worker	2–4	$80	$160–$320
**Diagnosis**	***a) Physical/developmental/medical assessment/examination***			
	Physician/pediatrician/developmental pediatrician/family doctorspecifically trained in FASD diagnosis	2–3	$160	$320–$480
	***b) Dysmorphology assessment***			
	Dysmorphologist and/or geneticist	1–2	$160	$160–$320
	***c) Neurobehavioural assessment***			
	Developmental Pediatrician	2–3	$160	$320–$480
	Psychologist	12–15	$100	$1,200–$1,500
	*Forms/questionnaires: cost per person (cost of kits are not included)*			$100
	Speech and Language Pathologist	2–3	$100	$200–$300
	*Forms/questionnaires: cost per person (cost of kits are not included)*			$25
	Occupational Therapist	2–3	$80	$160–$240
	*Forms/questionnaires: cost per person (cost of kits are not included)*			$25
	***d) Interviewing biological mother or foster parents or obtaining 2^nd^*** ***sources for maternal prenatal alcohol consumption confirmation***			
	Coordinator for case management (e.g., nurse, social worker)	2–4	$70	$140–280
***Total per person*** **		***26–39***		***$2,960–$4,370***
**General Support**	Secretarial/clerical	6–8	$25	$150–$200
**OVERALL per person** ***		**32–47**		**$3,110–$4,570**

* This stage is not limited to the individuals listed here.

** Excluding screening and referral, intake, and general support.

*** Including screening and referral, intake, diagnosis, and general support.

### Estimation of the Number of FASD Diagnosis per Year and the Associated Cost

A recent survey by Clarren and colleagues [22] reported that there were 44 clinics in 2011 that performed FASD multidisciplinary diagnostics in six provinces and one territory in Canada. The clinical capacity per year, which is the number of assessment slots available for diagnostic purposes (the maximum number of cases that can be assessed in any given year), was estimated as 2,288 for 2011 [22] (Table 2). However, in the majority of clinics, these slots are shared for the diagnosis of other complex developmental conditions, for example, autism spectrum disorder, that usually involve the same diagnostic team. As a result, there are fewer FASD diagnostic evaluations performed per year than the existing total number of diagnostic slots.

**Table 2:** Clinical capacity of FASD diagnosis in 2011, estimated number of people diagnosed with FASD per year (assuming a 50% and 70% FASD diagnosis rate), and the annual cost for FASD diagnosis in Canada, by available provinces and territory.

	Clinical Capacity (number of evaluation slots) in 2011 (Clarren et al. [22])	Estimated number of people diagnosed with FASD per year (based on a 50% diagnosis rate) Lower boundary	Cost of diagnosis peryear (assuming 50% diagnosed and cost per person = $3,110) Lower estimate	Cost of diagnosis peryear (assuming 50% diagnosed and cost per person = $4,570) Upper estimate	Estimated number of diagnosed people with FASD per year (based on a 70% diagnosis rate**) Upper boundary	Cost of diagnosis peryear (assuming 70% diagnosed and cost per person = $3,110) Lower estimate	Cost of diagnosis per year (assuming 70% diagnosed and cost per person = $4,570) Upper estimate
Alberta	387	194	$601,785	$884,295	271	$842,499	$1,238,013
British Columbia	765	383	$1,189,575	$1,748,025	536	$1,665,405	$2,447,235
Manitoba	198	99	$307,890	$452,430	139	$431,046	$633,402
New Brunswick	16	8	$24,880	$36,560	11	$34,832	$51,184
Ontario	512	256	$796,160	$1,169,920	358	$1,114,624	$1,637,888
Saskatchewan	280	140	$435,400	$639,800	196	$609,560	$895,720
Yukon	20	10	$31,100	$45,700	14	$43,540	$63,980
**Canada**	**2,288** *	**1,144**	**$3,557,840**	**$5,228,080**	**1,602**	**$4,980,976**	**$7,319,312**

* The final number is the full estimated capacity for FASD diagnosis in Canada as a whole, including additional slots from the rest of provinces and territories [22].

** Obtained from Clarren & Lutke [18].

Therefore, it was estimated that based on a capacity of 2,288 (i.e., the available number of evaluation slots) in all FASD clinics in Canada per year [22], assuming that all slots are completely filled, and assuming that about 70% of all individuals evaluated for FASD will be found to have an FASD [18] **,** one can calculate that there are at most approximately 1,602 cases of FASD diagnosed per year in Canada.

Using an assumption that only 50% of all individuals referred and evaluated in the multidisciplinary clinics are diagnosed with FASD, one can calculate that 1,144 cases (50%; lower boundary) of FASD are diagnosed per year in Canada. This estimate - 1,144 (50%; lower boundary) to 1,602 (70%; upper boundary) cases of FASD are diagnosed per year in Canada - was used to estimate the annual cost of FASD diagnosis in Canada. Therefore, it can be estimated that in Canada the total cost of diagnosing FASD ranges from $3.6 to $5.2 million (lower estimate), up to $5.0 to $7.3 million (upper estimate) per year (Table 2).

## Discussion

It could be argued that a large portion of the cost of FASD diagnostic services may be attributed to the need for the multidisciplinary approach. This approach could be considered to be too expensive and cumbersome for a high frequency disorder like FASD, where about one percent of all live births are affected and in some communities may even be much higher (up to 20%) [23]–[26] in Canada. However, it should be understood that those diagnosed using the multidisciplinary team approach benefit by being assessed by professionals that may not otherwise be seen (if they were to be assessed by a single practitioner). Further, individuals affected by FASD require a large amount of time be devoted to them in order to establish that their brain is damaged, the extent and specificity of its damage, and what interventions would be the most advantageous. In addition, many of the diagnostic clinics are involved in recommending and/or providing direct management, intervention, and services for these affected patients, services that would not be recognized as needed without the use of the diagnostic tests.

Given the fact that FASD is not widely recognized by health care practitioners [27], FASD is largely underdiagnosed. Coupled with this reality is the fact that, currently, the capacity of FASD clinics is low and the expertise needed to accommodate the demand is lacking. As mentioned, a recent survey by Clarren and colleagues [22] revealed that there were 44 clinics in 2011 that performed FASD multidisciplinary diagnostics in six provinces and one territory of Canada. The authors estimated that currently a seventeen-fold increase in FASD diagnostic capacity across Canada is needed in order to diagnose the number of FASD cases that currently exist (based on an FASD prevalence rate of 1%). Based on the above estimate, one can assume that the estimated cost for FASD diagnostic services in this study would be much higher, if the diagnostic capacity across Canada was not limited.

There are several other reasons to believe that the estimated cost of FASD diagnostic services in this study is most likely underestimated. To begin, the current cost estimate of FASD diagnosis per person includes the cost of the core team member only. However, additional team members may include addiction counselors, childcare workers, mental health workers, probation officers, psychiatrists, teachers, vocational counselors, nurses, neuropsychologists, and family therapists [10], all of whom would add additional costs. There may also be the need to consult with other professionals/community members. For example, when providing diagnostic assessments to Canadian Aboriginals, individual elders or elder councils may be consulted. Secondly, the cost of instruments (kits) was not included in the current cost estimate, even though this cost is likely significant. For example, as per expert opinion (personal communication with Kelly Nash, psychologist at The Hospital for Sick Children, Motherisk Program, Toronto) psychological instruments can add an additional cost of $330 to $500 per person, particularly in a clinic’s first year of operation. Thirdly, these cost estimates do not include facility costs (office space, medical records, or the other personal costs required to operate a medical facility), the cost of the time spent preparing for the assessments and writing the final reports, or the cost of the time spent by the diagnostic team in case conferences where the final diagnosis is confirmed. It is important to stress that a final diagnosis is a collaborative decision, one in which all members of the diagnostic team weigh in. This collaborative decision takes place during weekly/monthly diagnostic team meetings. According to the 2005 survey, conducted by the Canada Northwest FASD Research Network, among FASD clinical programs in Western and Northern Canada, the mean time directly spent with a patient was reported to be approximately the same as the time estimated by the experts in the current study. However, time for indirect care, which included chart review, team discussions, scoring of tests and note preparations, etc. was estimated to be approximately twice as high as that reported for direct patient care [18].

Finally, the estimated cost of diagnosis does not include the cost of completing an intervention plan and/or follow-up. It is important to point out that it would be unethical to identify an individual with an FASD and not provide them with the necessary referrals, education, resources, and services specific to their needs. Intervention plans should not only consist of identifying the deficits and impairments of the individuals with FASD, but also identifying the strengths of that individual. Therefore, an individualized intervention plan and follow-up must follow any diagnosis.

In addition, Canada is a multicultural country and thus, the entire diagnostic process must be culturally appropriate, sensitive, and use appropriate language. For this reason, the use of cultural interpreters may be required, and additional costs would likely be incurred in such cases.

Further, both the cost of negatively screened individuals and the cost of screening pregnant women are not included in the current total annual estimate. Such costs would not be incurred if maternal alcohol consumption while pregnant had a prevalence of zero. The inclusion of such costs would increase the current estimate.

The estimated cost of FASD diagnosis per person is comparable with the estimates obtained from the above-mentioned survey where the programs were asked to provide the average cost of an assessment [18]. Three of the public programs in this survey had roughly estimated their average cost of an evaluation for FASD per patient at $2,500 to $3,500 (mean and median: $3,000). Six non-profit private programs had an estimated per person cost ranging from $2,000 to $5,500 (mean and median: $3,500) [18].

There are several limitations of the current study. First, the estimated cost of FASD diagnosis is based on hourly wages. However, it should be noted that some members of the multidisciplinary team work on a salary basis, while others work on a sessional fee basis. This could affect the cost estimate in either direction depending on the level of compensation for salary and sessional fees relative to the hourly wages utilized here.

Second, not all individuals screened get referred for a full diagnostic assessment, not every person referred will show up for their evaluation, and not all individuals referred to a diagnostic assessment will receive an FASD diagnosis and therefore, not all individuals will proceed through the listed services in a linear fashion. As a result, the total cost of FASD diagnosis in Canada might be higher or lower.

Third, it was not possible to estimate the costs for the rest of the provinces and territories (Quebec, Newfoundland and Labrador, Nova Scotia, Prince Edward Island, Northwest Territories, and Nunavut) separately, since data on the clinical capacity of these provinces/territories were not available.

It is necessary to point out that the diagnostic schema could differ slightly from clinic to clinic, and therefore, variations are likely to exist in regard to the overall diagnostic process, for example, in the team members involved. This is likely true in remote areas, and small communities. Such variations will alter the overall cost per person. It is also important to highlight that the cost of diagnosing an individual with FASD in the general population, as presented here, will differ from the cost of diagnosing an individual with FASD in certain special populations, such as the prisoner population. For example, when diagnosing an individual in the criminal justice system additional costs are likely to be incurred, including the cost of transportation (transporting the prisoner or transporting the FASD-trained team members to the facility) and the cost of security/prison guards.

It should be highlighted that early detection of disease is not beneficial to the patient or his/her family if existing treatment is not offered or is not available [28]. Therefore, FASD diagnosis should not be just a label for a patient and his/her family, but should provide the basis for access to early interventions, treatment, family support and other preventive measures in order to minimize subsequent health, social and economic consequences of FASD. However, the counterargument is that it is unethical to deny a diagnosis for any patient just because a system of intervention is not fully developed. In fact, it has been shown that once a patient has been assessed to have FASD, this in itself drives the development of services [10]. Further, a cost-benefit analysis is needed to demonstrate the potential rates of return on expenditures made for the early diagnosis of FASD, which based on the long-term benefits of individuals with diagnosed FASD, described above, are expected to be high. The current study, which represents the first cost estimate of FASD diagnostic services in Canada, will facilitate such evaluation.
